# Reproductive outcome after in vitro fertilization in endometriosis – key factors and implications

**DOI:** 10.25122/jml-2024-0114

**Published:** 2024-03

**Authors:** Elena-Silvia Nadă, Ciprian Andrei Coroleucă, Cătălin Bogdan Coroleucă, Elvira Brătilă

**Affiliations:** 1 Department of Obstetrics and Gynecology, Carol Davila University of Medicine and Pharmacy, Bucharest, Romania; 2 Department of Obstetrics and Gynecology, Prof. Dr. Panait Sîrbu Clinical Hospital of Obstetrics and Gynecology, Bucharest, Romania

**Keywords:** endometriosis, infertility, in vitro fertilization, pregnancy, ovarian stimulation, AMH, anti-Mullerian hormone, BMI, body mass index, ESHRE – European Society of Human Reproduction and Embryology, FSH, follicle-stimulating hormone, GnRH, gonadotropin-releasing hormone, IVF, in vitro fertilization, MRI, magnetic resonance imaging

## Abstract

Endometriosis is a benign chronic disease with a major impact on a woman’s quality of life, mainly due to painful physical symptoms. Endometriosis is also a common cause of infertility caused by low ovarian reserve, distorted pelvic anatomy, and severe local inflammation with a direct negative impact on the quality of oocytes, embryos, and endometrium. We conducted a retrospective study between January 2019 and December 2023, including women with a history of surgery for endometriosis who underwent in vitro fertilization (IVF) to achieve pregnancy. Their reproductive outcome was compared with a group of patients with documented tubal obstruction. The aim of our study was to identify the factors associated with a positive impact on the pregnancy rate, specifically age, anti-Mullerian hormone (AMH), ovarian stimulation protocol, and types of gonadotropins used. We analyzed a group of 175 patients with endometriosis compared with 189 patients with tubal obstruction. The average age was similar between the two groups but with a difference in the average AMH value (1.63 ± 1.09 ng/mL vs. 2.55 ± 1.67 ng/mL). The most utilized ovarian stimulation protocol in both groups was the short gonadotropin-releasing hormone (GnRH) antagonist. The clinical pregnancy rate was 27.2% in the endometriosis group and 54.7% in the tubal obstruction group. Our study revealed that treatment with corifollitropin alfa in the endometriosis group was associated with a higher clinical pregnancy rate. AMH and age proved to be significant independent factors for the reproductive outcome.

## INTRODUCTION

Endometriosis is a chronic inflammatory disease defined by the presence of endometrial tissue outside the uterine cavity that has a detrimental impact on reproductive function. The incidence and prevalence of endometriosis are challenging to estimate, mainly due to underdiagnosis but also because patients are sometimes asymptomatic. In most studies, the incidence of endometriosis is around 10-15% among patients of reproductive age and can reach up to 45% in women with chronic pelvic pain and up to 50% in patients with associated infertility. The prevalence of this pathology among patients who underwent laparoscopy for infertility was up to 43.5%. More and more frequently, the diagnosis is established following the infertility consultation, the only sign in these cases being the failure to conceive [1–6].

The symptomatology in endometriosis is centered on pain, dysmenorrhea, dyspareunia, chronic pelvic pain, and, in most cases, associated with infertility [7]. Endometriosis is one of the leading causes of infertility, both primary and secondary, and it is mainly due to diminished ovarian reserve, altered pelvic anatomy, impaired folliculogenesis, hormonal imbalance, immune alterations with increased production of prostaglandins, proinflammatory cytokines and reactive oxygen species resulting in changes in all stages of reproduction– impaired ovulation, low oocyte quality, reduced sperm motility, alteration of tubal function and motility, low fertilization rate, direct toxic effect on the embryo, alteration of endometrial receptivity, and implantation failure [8–11].

Patients with endometriosis frequently turn to in vitro fertilization (IVF), most of the time, this being their only chance to get pregnant. Ovarian stimulation results and pregnancy rates in this category of patients are inferior to the results of IVF in patients with other causes of infertility [12]. The management of infertility associated with endometriosis is tailored according to the patient’s age, ovarian reserve, type and degree of endometriotic lesions, severity of symptoms, and reproductive plan of each patient.

According to the European Society of Human Reproduction and Embryology (ESHRE) guideline, IVF is recommended in patients with endometriosis and low ovarian reserve, documented tubal obstruction, associated male factor infertility, or failure to conceive naturally. No particular ovarian stimulation protocol or type of gonadotropin is recommended, and these can be chosen according to the preference of the fertility specialist [7].

This study aimed to evaluate the clinical characteristics of patients with a history of surgery for endometriosis and infertility and the outcomes of ovarian stimulation associated with a successful pregnancy compared to a control group of patients with tubal factor infertility.

## MATERIAL AND METHODS

We conducted a retrospective, observational, single-center study among women with a history of surgery for endometriosis and infertility who underwent IVF in order to achieve pregnancy. Their reproductive outcome was compared with a group of patients with documented tubal obstruction. The study was carried out between January 2019 and December 2023 at the Assisted Human Reproduction Department of Prof. Dr. Panait Sîrbu Clinical Hospital of Obstetrics and Gynecology in Bucharest, Romania.

Inclusion criteria involved the following: age 18–42 years, previous minimally invasive surgery for endometriosis (laparoscopic or robotic) for the study group, and documented tubal obstruction after hysterosalpingography or laparoscopy for the second group. Exclusion criteria included the following: IVF with donated oocytes, sperm donation or embryo donation, teratospermia on semen analysis, history of diseases with an impact on the reproductive outcome (cancer, cardiovascular or psychiatric disease), and incomplete data.

Before starting the IVF procedure, all patients underwent a series of tests in order to identify other possible causes of infertility. If hydrosalpinx was diagnosed, then tubal ligation or salpingectomy was performed in order to maximize pregnancy rates.

The ovarian stimulation protocol and the type and dose of gonadotropin were chosen according to the patient’s characteristics (age, anti-Mullerian hormone [AMH], antral follicle count, body mass index [BMI], and records of previous ovarian stimulations), but also according to the experience of the fertility specialist. After embryo transfer, luteal phase support was offered to all patients and included progesterone (vaginal, injectable, oral) in all cases, prenatal vitamins, corticosteroids, low-dose aspirin, or low molecular weight heparin in a personalized manner.

Statistical analysis was performed using IBM SPSS Statistics 25 and illustrated using Microsoft Office Excel/Word 2021. Quantitative variables were tested for distribution using the Shapiro-Wilk test and were expressed as means with standard deviations or medians with interpercentile ranges. Qualitative variables were expressed in absolute form or as a percentage and assessed using the Fisher's Exact Test/Pearson Chi-Square Test. Quantitative independent variables with non-parametric distribution were analyzed using the Mann–Whitney U test. Binomial logistic regression models were used to predict clinical pregnancy and were tested for model significance and validity, where the predictive performance of the variables was expressed as an odds ratio with 95% confidence intervals alongside the significance level.

## RESULTS

A total of 1391 patients underwent IVF procedures at the Assisted Human Reproduction Department of the Prof Dr Panait Sîrbu Clinical Hospital of Obstetrics and Gynecology between January 2019 and December 2023. After applying the inclusion and exclusion criteria, 175 patients with 298 embryo transfer cycles were included in the endometriosis group, and 189 patients with 303 embryo transfer cycles in the tubal obstruction group. The clinical characteristics of the groups are listed in Table 1.

**Table 1:** Clinical characteristics of the study groups

Clinical characteristic	Endometriosis	Tubal obstruction
Age	24 – 42 years	24 – 42 years
Dysmenorrhea	131 (74.9%)	105 (55.6%)
Dyspareunia	51 (29.1%)	13 (6.9%)
Chronic pelvic pain	64 (36.6%)	53 (28%)
Primary infertility	125 (71.4%)	76 (40.2%)
Smoking	52 (29.7%)	65 (34.4%)
AMH (average)	1.63 ± 1.09 ng/mL	2.55 ± 1.67 ng/mL
BMI (average)	22.97 ± 3.58 kg/m^2^	23.7 ± 3.44 kg/m^2^
Previous ectopic pregnancy	8 (4.6%)	62 (32.8%)
Previous miscarriage	48 (27.4%)	79 (41.8%)

**Figure 1:**
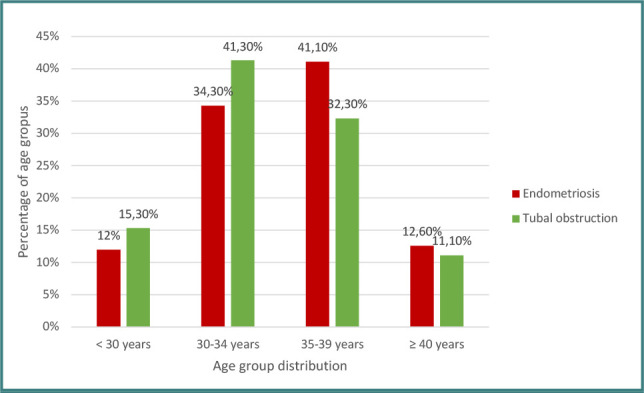
Age distribution of patients across study groups

The data in Figure 1 represent the distribution of patients according to age groups. In the endometriosis group, most patients were between 35 and 39 years old (41.1%%), and 46.3% of them were under 35 years old. The average age was 34.64 ± 3.82 years, the median was 35 years (interquartile range: 32–38 years). In the tubal pathology group, most patients were between 30 and 34 years old (41.3%), and 56.6% were under 35 years of age. The average age was 33.89 ± 4.08 years, and the median was 34 years (interquartile range: 31–37 years). The minimum age was 24 years, and the maximum was 42 years, which was identical in the two groups.

**Figure 2:**
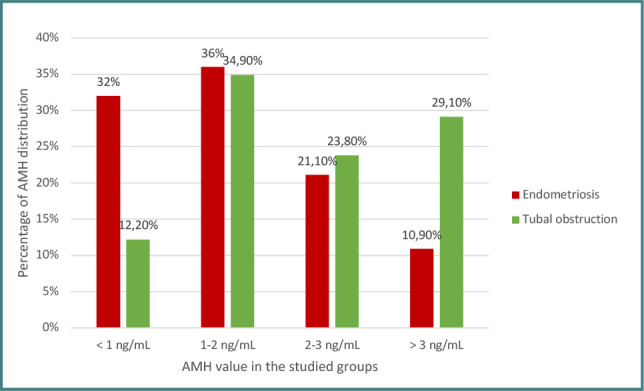
AMH value distribution in the study groups

The data in Figure 2 represents the distribution of patients based on their AMH value. In the endometriosis group, most had AMH levels between 1–2 ng/mL (36%) or below 1 ng/mL (32%). The average AMH was 1.63 ± 1.09 ng/mL, with a median of 1.47 ng/mL (interquartile range: 0.77–2.27 ng/mL). AMH values in this group ranged from 0.05 ng/mL to 4.98 ng/mL. Conversely, most patients in the tubal pathology group had AMH values between 1–2 ng/mL (34.9%) or above 3 ng/mL (29.1%). The average AMH was 2.55 ± 1.67 ng/mL, with a median of 2.05 ng/mL (interquartile range: 1.4–3.3). The minimum value for AMH was 0.31 ng/mL, and the maximum was 10.17 ng/mL.

The ovarian stimulation characteristics and reproductive outcome are listed in Table 2. Regarding ovarian stimulation outcomes, 12 patients (4%) in the endometriosis group and 4 patients (1.3%) in the tubal pathology group did not obtain any mature oocytes. The cancellation rate due to fertilization failure was 1.4% in the endometriosis group, while there were no cases in the tubal pathology group. No cases of hyperstimulation syndrome were recorded in any group.

**Table 2:** Ovarian stimulation characteristics and reproductive outcomes

	Endometriosis – 298 cycles	Tubal obstruction – 303 cycles
**Ovarian stimulation protocol**		
Short GnRH antagonist	263 (88.3%)	285 (94.1%)
Long GnRH agonist	30 (10.1%)	13 (4.3%)
Luteal phase	5 (1.7%)	5 (1.7%)
Dual trigger ovulation	126 (42.3%)	105 (34.7)
Number of stimulation days (average)	10.76 ± 1.69 days	10.44 ± 1.44 days
**Types of gonadotropins**		
Menotropin	253 (84.9%)	251 (82.8%)
Follitropin alfa	167 (56%)	151 (49.8%)
Follitropin beta	40 (13.4%)	49 (16.2%)
Follitropin delta	38 (12.8%)	67 (22.1%)
Corifollitropin alfa	28 (9.4%)	25 (8.3%)
Letrozole	28 (9.4%)	22 (7.3%)
Follitropin alfa + Lutropin alfa	22 (7.4)	13 (4.3%)
**Reproductive outcome**		
Mean no of oocytes	6.67 ± 4.03	10.59 ± 6.34
Mean no of mature oocytes	5.16 ± 3.14	8.41 ± 5.02
Mean no of embryos	4.04 ± 2.43	5.9 ± 3.47
Mean no of blastocysts	2.38 ± 2.17	4.41 ± 3.15
Biochemical pregnancy rate	46%	67.6%
Clinical pregnancy rate	27.2%	54.7%
Birth rate	22.9%	41.8%

GnRH, Gonadotropin-releasing hormone; No, number.

Further analysis of factors influencing clinical pregnancy revealed significant correlations. Both age and AMH values were significant predictors of clinical pregnancy in univariable and multivariable models (Table 3).

**Table 3:** Logistic regression models predicting clinical pregnancy in patients with endometriosis based on age and AMH values

Model/Parameter	Univariable		Multivariable	
	OR (95% CI)	*P*	OR (95% CI)	*P*
Age < 35 years	2.416 (1.3–4.494)	0.005	2.187 (1.144–4.18)	0.018
AMH < 1 ng/mL (Reference)	-	-	-	-
AMH: 1–2 ng/mL	2.324 (1.047–5.161)	0.038	2.382 (1.058–5.363)	0.036
AMH: 2–3 ng/mL	4.851 (1.966–11.969)	0.001	4.266 (1.7–10.704)	0.002
AMH > 3 ng/mL	2.406 (0.799–7.241)	0.118	2.133 (0.694–6.558)	0.186

In univariable models, patients under 35 years with endometriosis had a significantly increased (*P* = 0.005) chance of clinical pregnancy by 2.416 times (95% CI, 1.3–4.494). Furthermore, compared to patients with AMH values below 1 ng/mL, patients between 1–2 ng/mL had significantly 2.324 times higher odds of having a clinical pregnancy (95% CI, 1.047–5.161; *P* = 0.038). Similarly, patients with AMH levels between 2–3 ng/mL had 4.851 times higher odds of having a clinical pregnancy (95% CI, 1.966–11.969; *P* = 0.001).

In the multivariable model, patients under 35 years with endometriosis had a significantly increased (*P* = 0.018) chance of clinical pregnancy by 2.187 times (95% CI, 1.144–4.18) compared to those aged 35 or older. Compared to patients with AMH values below 1 ng/mL, patients with AMH values between 1–2 ng/mL had a significantly 2.382 times higher odds (95% CI, 1.058–5.363; *P* = 0.036) of having clinical pregnancy and patients with AMH values between 2–3 ng/mL had a significantly 4.266 times higher chance of clinical pregnancy (95% CI, 1.7–10.704; *P* = 0.002).

In univariable models, patients under 35 years with tubal pathology had a significantly increased likelihood of having a clinical pregnancy by 2,402 times (95% CI, 1,224–4,712; *P* = 0.011). Additionally, patients with AMH values above 3 ng/mL had a significantly 5.275 times higher chance of having a clinical pregnancy (95% CI, 1.68–16.557; *P* = 0.004) compared to patients with AMH values below 1 ng/mL. In the multivariable model, age and AMH value were significant and independent predictors of clinical pregnancy. Patients under 35 years with tubal pathology had a significantly increased chance of having a clinical pregnancy by 2,184 times (95% CI, 1,095–4,359; *P* = 0.027) compared to those aged 35 years or older. Compared to patients with AMH values below 1 ng/mL, patients with AMH values above 3 ng/mL had significantly 4,632 times (95% CI, 1,449–14,807; *P* = 0.010) higher odds of clinical pregnancy (Table 4).

**Table 4:** Logistic regression models for predicting clinical pregnancy using age groups and categories of AMH values in patients with tubal obstruction

Model/Parameter	Univariable		Multivariable	
	OR (95% CI)	*P*	OR (95% CI)	*P*
Age < 35 years	2.402 (1.224–4.712)	0.011	2.184 (1.095–4.359)	0.027
AMH < 1 ng/mL (Reference)	-	-	-	-
AMH: 1–2 ng/mL	1.769 (0.666–4.702)	0.253	1.653 (0.611–4.473)	0.323
AMH: 2–3 ng/mL	2.692 (0.911–7.954)	0.073	2.526 (0.840–7.598)	0.099
AMH > 3 ng/mL	5.275 (1.68–16.557)	0.004	4.632 (1.449–14.807)	0.010

**Figure 3:**
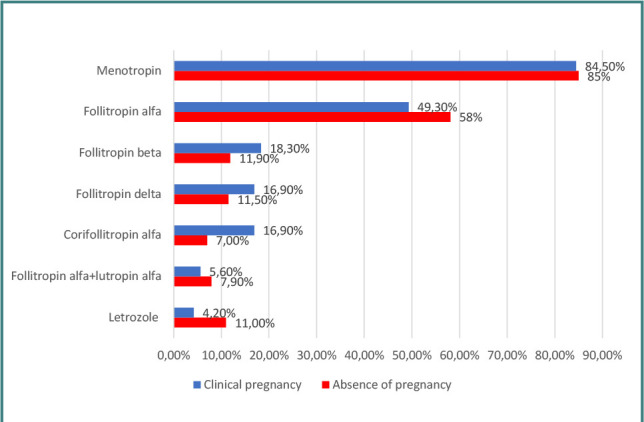
Distribution of gonadotropins in patients with endometriosis according to clinical pregnancy rate

The presence of dysmenorrhea, dyspareunia, chronic pelvic pain, smoking, and obesity (BMI>30 kg/m^2^) did not influence the presence of clinical pregnancy in any group (*P* >0.05) as determined by the Fisher’s and Mann-Whitney U tests. Similarly, the type of ovarian stimulation protocol had no statistically significant difference in the clinical pregnancy rate in either group according to Fisher’s Exact test (*P* = 0.485 in the endometriosis group and *P* = 0.581 in the tubal pathology group). Figure 3 presents the types of gonadotropins used in patients with endometriosis according to the presence of clinical pregnancy. Menotropin was the most commonly used gonadotropin in both groups, but it was only used in combination with other gonadotropins, not as a single medication. Letrozole was also used only in combination with gonadotropins in patients with benign breast tumors in order to minimize the total estradiol levels.

The differences between groups were not significant according to Fisher’s exact test (*P* >0.05) for most of the gonadotropins analyzed, except when assessing the association of clinical pregnancy with the administration of corifollitropin alfa in the endometriosis group and follitropin alfa+lutropin alfa in the tubal pathology group. When corifollitropin alfa was administered to patients with endometriosis, the clinical pregnancy rate was significantly higher (16.9% vs. 7%; *P* = 0.019). The univariable logistic regression models confirmed this, showing a 2.682-fold increased chance of clinical pregnancy with corifollitropin alfa (95% CI, 1,203–5,983; *P* = 0.016). This association remained significant in the multivariable model, with a 2,713-fold increase (95% CI, 1,186–6,207; *P* = 0.018) compared to cases where corifollitropin alfa was not used.

**Figure 4:**
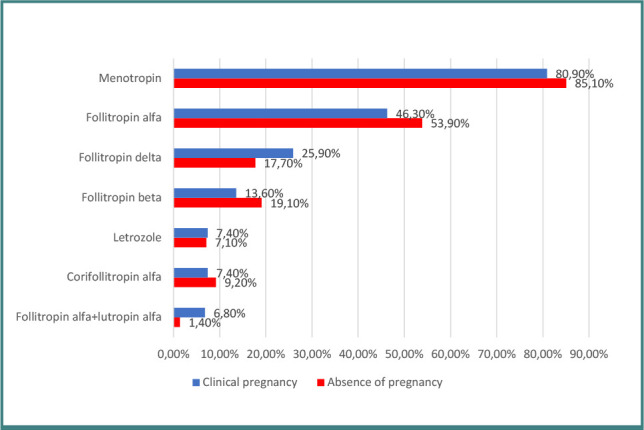
Distribution of gonadotropins in patients with tubal obstruction according to clinical pregnancy rate

Similarly, in the tubal pathology group, follitropin alfa+lutropin alfa resulted in a higher clinical pregnancy rate (6.8% vs 1.4%; *P* = 0.024) (Figure 4). The univariable analysis demonstrated a 5.063-fold increased chance of clinical pregnancy with this treatment (95% CI, 1,103–23,245; *P* = 0.037). In the multivariable model, treatment with follitropin alfa+lutropin alfa remained a significant factor in increasing the chance of clinical pregnancy by 5,520 times (95% CI, 1,177–25.9; *P* = 0.030) compared to cases where it was not used.

## DISCUSSION

Endometriosis is a benign disease with a major impact on a woman’s ability to achieve pregnancy both naturally and through IVF. Although Romania does not have a national registry or statistical data regarding the incidence of this pathology, our fertility department identified endometriosis as the cause of infertility in 12.6% of cases during the study period. Early diagnosis and optimal surgical treatment are valuable tools associated with successful infertility management. Despite the remarkable progress in imaging in terms of increased image quality and equipment availability, which have made possible a rapid and non-invasive diagnosis of endometriosis, except for superficial peritoneal implants, endometriosis remains one of the most underdiagnosed gynecological diseases [7,13]. Studies indicate an average diagnostic delay of 8-10 years from symptom onset, further emphasizing the negative impact on reproductive function [14–16]. Our study aimed to identify clinical characteristics that influence IVF pregnancy rates, focusing on age and AMH levels and a possible correlation between the ovarian stimulation protocol, the type of gonadotropin, and the pregnancy rate.

Ovarian reserve is negatively correlated with advancing age, even though there is considerable variation among women of the same age. Fertility reaches its peak between 20-29 years; after 30 years, it begins to decline gradually, and after 35 years, there is a sharp decline even in IVF pregnancies [17,18]. This trend is reflected in our study, where the majority of patients with endometriosis (53.7%) were over 35 years old, while the tubal pathology group was predominantly under 35 (56.6%). This advanced childbearing age supports the existence of diagnostic delays in endometriosis, delays associated with surgical interventions, and also due to the current trend in postponing the time at which a woman is planning to become pregnant. Age was an independent detrimental factor for the clinical pregnancy rate in both groups. Patients under 35 had a significantly higher chance of pregnancy than those over 35, regardless of the ovarian stimulation protocol, the types of gonadotropins used, and the number of oocytes or embryos obtained.

AMH is a glycoprotein produced by the granulosa cells of small preantral and antral follicles and is currently the main biomarker used to test ovarian reserve. It is an early marker of diminished ovarian reserve and correlates with response to ovarian stimulation but not pregnancy rate [17,19]. A value < 1ng/mL is associated with a poor response to stimulation, low oocyte quality, and low pregnancy rate [20]. Endometriosis, especially ovarian endometrioma, or a history of ovarian surgery for endometrioma are associated with diminished ovarian reserve. A 2018 meta-analysis evaluating AMH levels in unoperated patients with endometriotic ovarian cysts reported up to 26% lower AMH values in patients with endometriomas compared to a control group of patients with ovarian cysts of other etiology and patients without ovarian pathology [21].

All patients in our study underwent minimally invasive surgery for endometriosis, and most of them had a postoperative AMH value between 1–2 ng/mL (36%) or below 1 ng/mL (32%). The average AMH value was statistically significant between the two groups (1.63 ± 1.09 ng/mL vs. 2.55 ± 1.67 ng/mL). The AMH value positively correlated with the clinical pregnancy rate in our study and proved to be an independent statistically significant factor in obtaining a clinical pregnancy in both groups. For this reason, special care must be taken when operating on ovarian endometriomas in order not to damage an already affected ovary, and all endometriotic lesions should be excised in a single surgical intervention, ‘one-stop shop surgery’.

Endometriosis is mainly associated with painful symptoms. This was also the case in this study; more patients with dysmenorrhea, dyspareunia, and chronic pelvic pain were encountered in the endometriosis group compared with the tubal pathology group. However, these factors did not influence the pregnancy rate.

Smoking and obesity are well-known factors that negatively influence conception, folliculogenesis, implantation, and IVF outcomes. It was demonstrated that smokers have a lower AMH level and number of retrieved oocytes, a higher rate of canceled cycles, a thicker zona pellucida, and lower implantation rates than nonsmokers [22]. In our study, smoking and obesity were not found to have a deleterious effect on the clinical pregnancy rate.

The number of mature oocytes and embryos obtained from patients with endometriosis was significantly lower than those with tubal pathology. Endometriosis amplifies oxidative stress at the follicular level, and the production of reactive oxygen species induces meiotic abnormalities and chromosomal instability, thus altering the quality of the oocyte by thickening the zona pellucida, which makes fertilization, and later implantation, difficult. Studies in this area are somewhat limited for ethical reasons, but recent studies have evaluated spindle morphology as a marker of oocyte quality. A 2014 study found that oocytes retrieved from women with endometriosis had a higher percentage of spindle abnormalities compared to controls without endometriosis (66.7% vs. 16%) and a higher rate of apoptosis (80% vs. 22.2%) [11,23,24]. These morphological alterations in the oocyte indicate a lower number of embryos obtained due to failed fertilization and, subsequently, a lower clinical pregnancy rate in patients with endometriosis. This is consistent with our research, with the clinical pregnancy rate in the endometriosis group being much lower than in the tubal factor infertility group (27.2% vs. 54.7%).

Regarding the ovarian stimulation protocol, both short GnRH antagonists and long GnRH agonists are equally recommended in endometriosis according to the ESHRE recommendations [7]. In our study, we mostly used the short GnRH antagonist protocol in both groups (88.3% and 94.1%) due to its advantages over the long GnRH agonist protocol: shorter stimulation period, less ovarian inhibition, and lower incidence of hyperstimulation syndrome. Most studies looking at the difference between the long agonist and short antagonist protocol did not observe a statistically significant difference in pregnancy rate or birth rate regardless of disease stage, and that was also the case in our study [25,26].

An important finding in our research was the statistically significant higher pregnancy rate associated with the use of corifollitropin alfa compared with other gonadotropins in patients with endometriosis. Since the choice of gonadotropin is one of the few modifiable factors in the IVF process, selecting the appropriate type and dose from the outset is of the utmost importance. Corifollitropin alfa has a longer serum half-life than conventional recombinant FSH, and a single dose administered at the beginning of the stimulation period is sufficient for seven days of multiple follicular development, thus reducing the total number of injections and increasing patient comfort. Some studies indicate a higher number of retrieved oocytes and a higher pregnancy rate when corifollitropin alfa is used in poor responder patients, while other studies found no difference in pregnancy rates but only in the number of retrieved oocytes [27–29].

Our research has certain limitations that should be considered when interpreting the results. Firstly, the study is retrospective and conducted in a single specialized center of assisted human reproduction. We consider that a particular limitation is that we did not take into account embryo quality as a plausible cause of the low pregnancy rate in the endometriosis group. All types of embryos were included in the study because we considered that a low-quality embryo indirectly reflects the low quality of the oocytes, a fact demonstrated in endometriosis. To minimize the confounding effects of male factor infertility on fertilization failure, implantation failure, or embryo quality, we excluded patients with teratospermia based on semen analysis. However, future research should focus on analyzing the correlation between embryo quality and pregnancy rates in patients with endometriosis to provide a more comprehensive understanding of their reproductive outcome.

## CONCLUSION

In recent years, endometriosis has emerged as a significant public health concern, evidenced by the growing number of patients seeking specialized care and assisted reproductive technologies, and thus constitutes a current topic of interest to the medical community. Our study revealed that patients with endometriosis have a significantly lower chance of pregnancy obtained by IVF than those with tubal factor infertility. AMH and advancing age were identified as significant independent factors influencing the clinical pregnancy rate in both groups. The use of corifollitropin alfa for ovarian stimulation could be a valuable option for patients with endometriosis as it was associated with a statistically significant higher clinical pregnancy rate.

## Data Availability

Further data is available from the corresponding author upon reasonable request.
